# Associations between Diabetes-Specific Medication Regimen Complexity and Cardiometabolic Outcomes among Underserved Non-Hispanic Black Adults Living with Type 2 Diabetes Mellitus

**DOI:** 10.3390/pharmacy12030083

**Published:** 2024-05-26

**Authors:** Cheryl Wisseh, Edward Adinkrah, Linda Opara, Sheila Melone, Emem Udott, Mohsen Bazargan, Magda Shaheen

**Affiliations:** 1 Department of Clinical Pharmacy Practice, School of Pharmacy & Pharmaceutical Sciences, University of California, Irvine, CA 92697, USA; 2 Department of Family Medicine, College of Medicine, Charles R. Drew University of Medicine and Science (CDU), Los Angeles, CA 90059, USA; edwardadinkrah@cdrewu.edu (E.A.); mohsenbazargan@cdrewu.edu (M.B.); 3 Adult and Children’s Psychiatric Outpatient Clinic, Fresno County Department of Behavioral Health, Fresno, CA 93702, USA; lopara@fresnocountyca.gov; 4 Health and Wellness Center, Walmart Pharmacy, Bakersfield, CA 93307, USAeludott.s03141.us@wal-mart.com (E.U.); 5 Department of Family Medicine, David Geffen School of Medicine, University of California, Los Angeles (UCLA), Los Angeles, CA 90095, USA; 6 Department of Internal Medicine, College of Medicine, Charles R. Drew University of Medicine and Science (CDU), Los Angeles, CA 90059, USA; magdashaheen@cdrewu.edu

**Keywords:** health disparities, type 2 diabetes, diabetes management, medication regimen complexity, medication adherence, cardiometabolic outcomes

## Abstract

Type 2 diabetes mellitus (T2DM) management and glycemic control in underserved non-Hispanic Black adults presents with multifaceted challenges: balancing the optimal complexity of antihyperglycemic medications prescribed, limited medication access due to socioeconomic status, medication nonadherence, and high prevalence of cardiometabolic comorbidities. This single-center, cross-sectional, retrospective chart analysis evaluated the association of Medication Regimen Complexity (MRC) with cardiometabolic outcomes (glycemic, atherogenic cholesterol, and blood pressure control) among non-Hispanic Black adults with type 2 diabetes. Utilizing 470 independent patient electronic health records, MRC and other covariates were examined to determine their associations with cardiometabolic outcomes. Chi-square tests of independence and multiple logistic regression were performed to identify associations between MRC and cardiometabolic outcomes. Our findings indicate significant negative and positive associations between MRC and glycemic control and atherogenic cholesterol control, respectively. However, there were no associations between MRC and blood pressure control. As diabetes MRC was shown to be associated with poor glycemic control and improved atherogenic cholesterol control, there is a critical need to standardize interdisciplinary diabetes care to include pharmacists and to develop more insurance policy interventions that increase access to newer, efficacious diabetes medications for historically marginalized populations.

## 1. Introduction

According to the National Diabetes Statistics Report, when compared to non-Hispanic White persons (NHWs) at 13.6%, the total percentage of non-Hispanic Black persons (NHBs) that have diagnosed and undiagnosed diabetes is 17.4%, even though NHBs make up only 13.6% of the United States population [1,2]. Non-Hispanic Black persons also face unequal outcomes in comparison to NHWs regarding diabetes related risk factors and macrovascular complications. In 2021, the percentage of diagnosed hypertension among NHBs was 1.2 times that of NHWs and the percentage of obesity among NHBs was almost 1.4 times that of NHWs [3,4]. Although the percentage of NHWs with high cholesterol in was 1.2 times that NHBs with high cholesterol, the percentage of NHBs that died from heart disease was almost 1.3 times that of NHWs [5,6]. Furthermore in 2020, the age-adjusted stroke death rate was higher among NHBs (56.8 per 100,000) when compared to NHWs persons (37.1 per 100,000) [7]. Health care disparities are also present among NHBs regarding medication use for diabetes management. A cross-sectional study that assessed the trends in longitudinal use of antihyperglycemic, antihypertensive, and antihyperlipidemic medications in US adults with diabetes revealed that non-Hispanic Black NHBs participants’ use of antihyperglycemic medications was more likely to be inconsistent than continuous when compared to non-Hispanic White participants [8]. Poor medication adherence has also been linked to adverse health outcomes among NHBs. More specifically, low-income racially and ethnically minoritized patients with diabetes and poor medication adherence often have higher risks of diabetes morbidity and mortality when compared to white patients and patients with high socioeconomic status (SES) [9]. As various health disparities exist for NHBs regarding diabetes outcomes and medication adherence, it is crucial to understand the contributory factors to medication regimen nonadherence and its association with diabetes outcomes.

The pathophysiological mechanisms of diabetes mellitus are multifaceted and pharmacological management often requires providers’ prescription and patients’ use of several medications from different anti-hyperglycemic therapeutic classes. This is evidenced by the fact that 8 of the top 25 drugs with the highest expenditures in the United States market in 2021 were diabetes medications [10]. Furthermore, diabetes mellitus is one of several component conditions that comprises cardiovascular-kidney-metabolic syndrome (CKM). A disproportionately high burden of cardiovascular disease is attributed to CKM as it affects myocardial function, atherogenesis, and vascular integrity, which in turn increases the risks of coronary heart disease and stroke [11]. Thus, increased prescribing of multiple antihyperglycemic and cardioprotective medications to manage hyperglycemia, delay macrovascular and microvascular complications, improve health-related quality of life, and reduce diabetes mortality is certainly justified. However, this can also add a further layer to medication regimen complexity, which in turn may have a negative effect on medication adherence, glycemic control, and its related outcomes. In one study that consisted of African American adults in South Los Angeles, patient level medication regimen complexity (MRC) was measured by the validated Medication Regimen Complexity Index (MRCI) Microsoft (MS) Access database tool and was associated with an increased odds of medication nonadherence [12,13]. In addition to medication nonadherence, studies have also shown that patient-level MRC is associated with glycemic and blood pressure control [14,15,16].

Diabetes medication regimen complexity measures the complexity of all the antihyperglycemic medications that a patient is taking and its association with high hemoglobin A1c (HbA1c), and glycemic control has also been demonstrated in several studies [14,15,17,18]. However, there is a paucity of literature on the association between diabetes MRC and cardiometabolic outcomes such as atherogenic cholesterol control (cholesterol that promotes plaque formation in the arteries, such as low-density lipoprotein-cholesterol, is atherogenic) and blood pressure control. Although studies have evaluated the relationship between medication regimen complexity and glycemic control in diverse populations, the population in this study is a cohort that is composed solely of non-Hispanic Black adults living with diabetes in South Los Angeles—a geographical area that bears an excess burden of adverse effects from the inequitable distribution of the social determinants of health [19]. The inequitable distribution of social determinants of health is often a cause and driver of health and health care disparities and has been associated with poor glycemic outcomes among historically disenfranchised and marginalized populations [20]. Understanding medication regimen complexity for this population is a critical step to the improvement and maintenance of glycemic control, diabetes self-management, and reduction of health disparities. The objective of this study was to evaluate the association of medication regimen complexity with cardiometabolic outcomes (glycemic, atherogenic cholesterol, and blood pressure control) among non-Hispanic Black adults living with type 2 diabetes mellitus (T2DM). 

## 2. Methods

### 2.1. Design and Setting 

This study was a single-center, cross-sectional, retrospective chart review of patients receiving primary care at To Help Everyone Health and Wellness Center (T.H.E Clinic). The review period was 1 January 2010–30 June 2021. T.H.E Clinic is a federally qualified health care center in South Los Angeles that serves predominantly low-income patients across the lifespan that identify as non-Hispanic Black or Latino or are immigrants of African and/or Latin American ancestry.

### 2.2. Study Population

Patients that were 18 years and older, diagnosed with type 2 diabetes (based on ICD-9 or ICD-10 diagnosis code E.11*), and self-identified as non-Hispanic Black were included in the analysis. All participants in the analysis were placed into one of three diabetes mellitus severity categories. Diet-controlled diabetes was defined as not taking antihyperglycemic medications, uncomplicated diabetes was defined as taking diabetes medications and no end organ damage, and complicated diabetes was defined as a diagnosis with end organ damage [21]. End organ damage was defined as having one of the following: coronary heart disease, myocardial infarction, heart failure, cerebrovascular disease, peripheral vascular disease, neuropathy, nephropathy, retinopathy, or requiring dialysis [21,22,23,24]. 

### 2.3. Data Collection and Quality Control 

Two authors, (S.M. and E.U.), were trained extensively by the principal investigator (C.W.) on data extraction from the electronic medical record. The duration of the data extraction period was 6 months. The data were checked biweekly for accuracy and completeness by the principal investigator. Both data collectors were Doctor of Pharmacy candidates at the time of data collection. One author (L.O.) was trained extensively on analysis of medication regimens with the Microsoft Access Version 1.0 medication regimen complexity (MRC) electronic data capture tool that was used by the co-investigator (E.A.) and principal investigator (C.W.) in prior work [12,25]. The patient-level and diabetes-specific MRC indices were calculated for all patients included in the study solely by this author (L.O.). A team of pharmacists and one physician met periodically to discuss and resolve (by consensus) patient regimen cases that did not easily conform to the instructions provided with the medication regimen complexity electronic data capture tool (L.O., C.W., and E.A.). The principal investigator reviewed the medication regimen complexity index scoring record of all patients included in the study as a final check. 

### 2.4. Outcome Measures

Social determinants of health, medical history, medication regimens, and clinical outcome data were obtained from patients’ electronic medical records. Diabetes-specific and patient-level MRC were the predictor variables, and the covariates included: age, sex, insurance status, employment status, alcohol use status, smoking status, federal poverty level, World Health Organization (WHO) body mass index categories, and the Charlson Comorbidity index (CCI) [21,26]. The primary outcome was glycemic control, and the secondary outcomes were atherogenic cholesterol control and systolic and diastolic blood pressure control. More specifically, each outcome variable was operationalized as a binary outcome: controlled cardiometabolic outcome (success) and uncontrolled cardiometabolic outcome (failure). For glycemic control, a hemoglobin A1c (HbA1c) measure less than 7% was defined as “controlled” and an HbA1c greater than or equal to 7% was defined as “uncontrolled” [27]. For atherogenic cholesterol control, a low-density lipoprotein cholesterol (LDL-C) measure less than 100 mg/dL was defined as “controlled” while an LDL-C measure greater than or equal to 100 mg/dL was defined as “uncontrolled” [28]. Finally, for blood pressure (BP) control, systolic and diastolic blood pressure measures less than 140 mm Hg and 90 mm Hg, respectively, indicated “controlled” BP and a systolic blood pressure (SBP) and diastolic blood pressure (DBP) measure greater than or equal to 140 mm Hg and 90 mm Hg were defined as “uncontrolled” BP, respectively [29]. 

### 2.5. Medication Regimen Complexity 

The medication regimen complexity index (MRCI) is derived from a 65-item assessment tool that has been used in previous studies to objectively quantify the complexity of patient medication regimens based on the quantity of medications taken, dosage form(s), dosage frequency, and additional instructions [12,30]. The form consists of 3 sections: dosage form/drug administration route, dosing frequency, and additional directions. The final index is generated by the additions of the scores from all 3 sections. The medication regimens within the patient electronic health record were analyzed for both the patient-level and diabetes-specific MRC. The patient-level MRC includes all prescription medications, over-the-counter medications, and herbal supplements as documented in the patient electronic medical record. The diabetes-specific MRC (diabetes MRC) includes antihyperglycemic medications in the following pharmacological categories: biguanides, sodium-glucose cotransporter-2 inhibitors, glucagon-like peptide 1 agonists, dipeptidyl peptidase-4 inhibitors, thiazolidinediones, sulfonylureas, insulins, meglitinides, alpha-glucosidase inhibitors, amylin mimetics, combination oral antihyperglycemics, and combination insulin products. 

### 2.6. Medication Regimen Complexity Index Stratification Categories

The patient-level and diabetes-specific medication regimen complexity index (MRCI) stratification categories were based on the tertiles within the data and were designated as low, moderate, or high. The patient-level MRCI category cutoffs were low: ≤11.5; moderate: 11.6–31.4; high: ≥31.5; and the diabetes-specific MRCI cutoffs were low: ≤3; moderate: 4–8; high: ≥9. 

### 2.7. Charlson Comorbidity Index

The Charlson Comorbidity Index (CCI) is a comorbidity assessment tool for categorizing patient comorbidities. Comorbidities are weighted (range 1–6) and the sum of the weighted scores results in the final comorbidity score [21]. The comorbidities include congestive heart failure, peripheral vascular disease, cerebrovascular accident or transient ischemic stroke, dementia, chronic obstructive pulmonary disease, connective tissue disease, peptic ulcer disease, liver disease, diabetes mellitus, hemiplegia, moderate to severe chronic kidney disease, solid tumor, leukemia, lymphoma, and AIDS [21]. The highest attainable score is 37 and there is a positive association with higher score and mortality [31]. 

### 2.8. Sample Size Calculation and Hypotheses 

The sample size was calculated for a single population with a dichotomous outcome. The primary outcome was controlled HbA1c, and the secondary outcomes were controlled atherogenic cholesterol (LDL-C) and blood pressure. The final sample size was estimated to be 380 independent patient medical records. The hypotheses (H) for each outcome were as follows: H1: poor glycemic control will be positively associated with high medication regimen complexity; H2: poor atherogenic cholesterol control will be positively associated with high medication regimen complexity; H3: poor blood pressure control will be positively associated with high medication regimen complexity.

### 2.9. Statistical Analyses

The study population’s sociodemographic, clinical and medication regimen characteristics, and cardiometabolic outcomes were analyzed with descriptive statistics and their measures are reported as numbers (percentage) or means and standard deviations. The Chi-Square test of independence was used to examine the associations among categorical variables: patient-level MRC categories and diabetes-specific MRC categories; MRC categories and cardiometabolic outcomes; diabetes-specific MRC category and diabetes mellitus severity. A multiple logistic regression was performed between the primary outcome (glycemic control) and secondary outcomes (atherogenic cholesterol and blood pressure control), the predictor variable, and covariates. The results are reported as adjusted odds ratio (AOR) and 95% confidence interval (CI). The significance was set with a two-sided alpha of <0.05. All data were analyzed with IBM SPSS, v27 (Armonk, NY, USA). 

### 2.10. Institutional Review Board 

The study protocol was approved by the institutional review boards of Charles R. Drew University of Medicine and Science and the University of California, Irvine.

## 3. Results

### 3.1. Population Characteristics

A total of 470 patients who met the inclusion criteria were included in the final analysis. Table 1 depicts the sociodemographic characteristics of the study population. The mean age of participants was 60.3 years (SD ± 12.0) and 61.3% of the population were women. While a majority of the participants lived below the federal poverty line (79.8%), a majority of the participants were insured (92.1%). Medicare and Medicaid were the main source of insurance for almost 90% of the participants. Less than 1% of the participants self-reported housing insecurity and 66% were unemployed. A majority of the participants reported their smoking status as “never” (62.1%) and 62% did not use alcohol. 

Table 2 shows that 1 in 3 participants had complicated diabetes. Most of the participants had comorbid hypertension (83.4%), dyslipidemia (70%), or obesity (55.2%). While the mean blood pressure of the study population was at goal (SBP: 135.9 mm Hg and DBP: 81.2 mm Hg), both mean HbA1c and LDL-C were not at goal (8.4% and 109 mg/dL, respectively).

This correlates with Table A1, whereby more participants lacked glycemic and atherogenic cholesterol control and more participants had controlled blood pressure. The mean number of medications taken overall was 8.5 and the mean patient-level MRCI was 22.4. In alignment with the mean, the medication regimens of 81% of participants were designated as polypharmacy. The mean number of diabetes medications taken was 1.8 and the mean diabetes-specific MRCI was 1.8. As depicted in Table 3, the top 3 antihyperglycemic pharmacological classes that were utilized by the participants were biguanides (66.4%), second generation sulfonylureas (33.8%), and long-acting insulin (15.5%).

### 3.2. Associations between Medication Regimen Complexity and Cardiometabolic Outcomes

**Figure 1:**
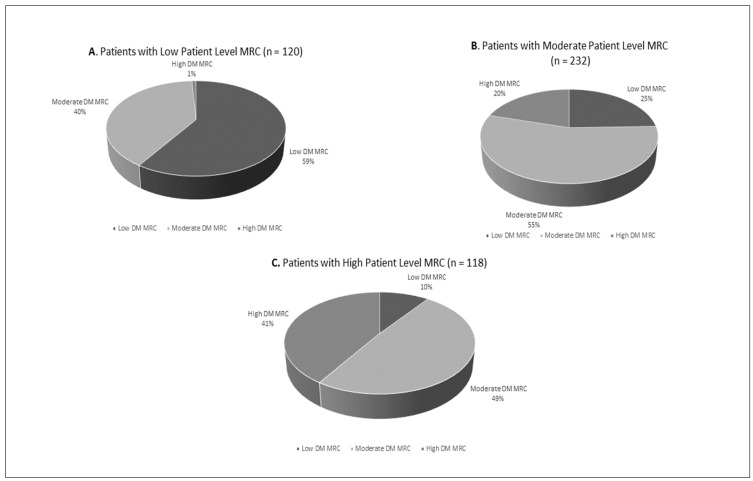
Patient-level medication regimen complexity (MRC) categories by proportion of diabetes-specific MRC categories (Pearsons Chi-Square *p* < 0.001).

The majority of participants with low patient-level MRC also had low diabetes-specific MRC (59%), shown in Figure 1A. In a descending trend, 40% of these patients had moderate diabetes-specific MRC and 1% had high diabetes-specific MRC. Similarly, most patients with moderate patient-level MRC also had moderate diabetes-specific MRC (55%). This relationship was not observed with patients with high patient-level MRC as most patients had moderate diabetes MRC (Figure 1B,C). 

Both patient-level and diabetes-specific MRC were associated with glycemic and atherogenic cholesterol control across all categories of MRC (low, moderate, high). More specifically, as shown in Table 4, glycemic control differed significantly across diabetes-specific MRC groups with the highest level of control occurring in those with low diabetes-specific MRC (67.7%) and the lowest level of control occurring in those with high diabetes-specific MRC (12.0%). Interestingly, atherogenic cholesterol control differed significantly across patient level and diabetes-specific MRC groups. The lowest level of atherogenic cholesterol control occurred in those with low patient-level and diabetes-specific MRC and highest level of atherogenic cholesterol control occurred in those with high patient-level (56.7) and moderate diabetes-specific (51.8) MRC, respectively. The patient level and diabetes-specific MRC were not associated with blood pressure control (SBP, DBP) at any level of MRC. Data that were not statistically significant were not reported. 

Table A2 depicts the associations between diabetes-specific MRC category and diabetes mellitus severity. All participants with diet-controlled diabetes had low diabetes-specific MRC (100%). Moderate diabetes-specific MRC was of highest prevalence across both groups of participants with uncomplicated (50.9%) and complicated diabetes (55.1%) and low diabetes-specific MRC was least prevalent in those with complicated diabetes mellitus (18.4%). 

Adjusting for the other independent variables, when compared to participants with low diabetes-specific MRC participants with moderate diabetes-specific MRC were 5 times more likely to lack glycemic control and participants with high diabetes-specific MRC were almost 24 times more likely to lack glycemic control (*p* = 0.000) (Table 5). Participants with moderate diabetes-specific MRC were 50% less likely to have atherogenic cholesterol that was not at goal (*p* = 0.036). Furthermore, the use of alcohol, pre-obesity, and Class 2 and 3 obesity statuses were independently associated with lack of atherogenic cholesterol control (*p* = 0.027, *p* = 0.014, *p* = 0.016, respectively).

While high patient-level MRC was associated with higher odds of uncontrolled HbA1c, the association between moderate patient-level MRC and uncontrolled HbA1c did not reach statistical significance (Table 6). In a similar fashion to diabetes-specific MRC, participants with moderate and high patient-level MRC were 61% and 77% less likely to have atherogenic cholesterol that was not at goal, respectively. Pre-obesity, and class 2 and 3 obesity classifications were also independently associated with uncontrolled atherogenic cholesterol.

## 4. Discussion

In this study, a validated MRCI MS Access database tool was utilized to quantify patient-level and diabetes-specific MRC among Non-Hispanic Black adults with T2DM who were patients at a Federally Qualified Health Care center in South Los Angeles [12]. Associations between diabetes-specific and patient-level MRC and glycemic, atherogenic cholesterol, and blood pressure control were also evaluated. We found that both diabetes-specific and patient-level MRC were associated with glycemic and atherogenic cholesterol control across all MRC categories (low, moderate, high). However, diabetes-specific and patient-level MRC were not associated with blood pressure control. The results also indicate that diabetes-specific and patient-level MRC predict both glycemic and atherogenic cholesterol control and that pre-obesity and class 2 and 3 obesity statuses are independently associated with atherogenic cholesterol control. To our knowledge, this is the first study to evaluate the relationship between cardiometabolic outcomes and medication regimen complexity within a historically racially minoritized population in the US. 

In our study, there was a significant association between patient level and diabetes-specific MRC and glycemic control at all MRC levels (low, moderate, high). More specifically, moderate and high diabetes-specific MRC were independently associated with lack of glycemic control. This is consistent with the findings of Yeh et al. and Ab Rahman et al., who demonstrated that high- and moderate-diabetes MRC were associated with lower odds of HbA1c goal attainment [15,17]. Moreover, high patient-level MRC was also associated with higher odds of uncontrolled HbA1c, which also aligned with the findings of Ab Rahman et al. [15]. Although diabetes distress (DD) was not an outcome measured in our study, it has been linked to diabetes MRC, HbA1c, and medication nonadherence. One study that evaluated the associations between diabetes MRC and diabetes-related distress revealed that although the diabetes duration, hypertension and dyslipidemia burden, and sociodemographic characteristics among the diabetes MRC groups were similar, there was a significant difference in HbA1c, with higher HbA1c occurring in the high and moderate diabetes MRC group than in the low diabetes MRC group (*p =* 0.006) [32]. The prevalence of diabetes distress and high diabetes distress scores were also significantly higher in the high diabetes MRC group when compared to the moderate and low diabetes MRC groups (*p* = 0.006; *p* = 0.009) [32]. Furthermore, Hessler et al. and Cummings et al. found independent associations between high HbA1c, greater regimen distress, and poor medication adherence [33,34]. Thus, patients with high diabetes MRC might experience higher levels of distress associated with their medications and the other self-management requirements of living with and managing diabetes on a day-to-day basis. These aspects might in turn, contribute to medication nonadherence. 

There was a significant association between patient-level and diabetes-specific MRC and atherogenic cholesterol control at all MRC levels (low, moderate high). Regression analyses showed that moderate diabetes MRC, moderate patient-level MRC, and high patient-level MRC were associated with higher odds of atherogenic cholesterol control. These results seem to suggest that moderate and high MRC provide a protective advantage for atherogenic cholesterol control. This phenomenon might be explained by the utilization of statins (55%) and the biguanide antihyperglycemic pharmacological class (66.4%) in the study population. It is well known that the primary treatment indication of the statin pharmacological class is dyslipidemia and that medications in this class reduce LDL-C. However, metformin is a drug within the biguanide class and a mainstay and first-line oral medication in the treatment of prediabetes and type 2 diabetes. Mechanistically, it reduces hepatic glucose production, decreases intestinal absorption of glucose, and improves insulin sensitivity in peripheral tissues [35]. Hu et al. revealed a new mechanism of action by which metformin lowered low-density lipoprotein cholesterol levels. More specifically, metformin reduced the translocation of the carbohydrate-responsive element-binding protein (ChREBP) from the cytosol to the nucleus [36]. During normal pathophysiological function, ChREBP acts as a glucose switch (sensing elevated glucose levels activates its translocation) and leads to the expression of target genes such as proprotein convertase subtilisin/kexin type 9 (PCSK9) [36]. The role of PCSK9 is to bind the low-density lipoprotein [cholesterol] receptor (LDLR) on the hepatocyte surface, which then leads to the degradation of the LDLR [36]. Lower LDLR levels result in higher circulating LDL-C (atherogenic cholesterol) levels in the blood. Thus, metformin reduces hepatocyte intracellular glucose production, which turns off the glucose sensing of ChREBP, which then reduces PCSK9 transcription. The significance of reduced PCSK9 is increased LDLR on the hepatocyte surface and lower circulating LDL-C levels. 

Antihyperglycemic medications that are considered more novel when compared to metformin may also have effects beyond improved hyperglycemia. Sodium-glucose Cotransporter 2 inhibitors (SGLT2i), glucagon-like peptide-1 receptor agonists (GLP-1RA), and dipeptidyl peptidase-4 inhibitors (DPP-IVi) have all been evaluated in pre-clinical and clinical studies to further elucidate their role in lipid profile changes [37]. The evidence has been promising for two of these drug classes, as SGLT2i and GLP-1RA have been recommended in clinical guidelines as first-line agents, with or without metformin, for concomitant cardiovascular risk reduction in patients with diabetes and compelling cardiovascular conditions [38,39]. However, only 9.4%, 3.8%, and 1% of patients in this study were taking DPP-IVi, SGLT2i, and GLP-1RA, respectively. Thus, any reduction in LDL-C may likely be due to the use of statins and metformin through the aforementioned mechanism. The lower utilization of these medications in the study population may likely be due to the long review period for the study and medication costs. Newer medications in the SGLT2i and GLP-1RA pharmacological classes first entered the market, several landmark clinical trials were published, and clinical guidelines were updated several times during the study’s review period (2010–2021). Moreover, given that 80% and 91% of patients in this study live below the federal poverty line and have government-funded insurance, it is likely that formulary constraints may have limited their access to the newer medications. We did not collect data regarding the diet type and physical activity levels of the participants. However, only 4.5% of participants had diet-controlled diabetes and 55% of the participants were classified as obese. Thus, lifestyle modifications such as dieting, and exercise are less likely explanations to support the associative link between atherogenic cholesterol control and medication regimen complexity. 

Pre-obesity and class 2 and 3 obesity statuses were independently associated with lack of atherogenic cholesterol control and the independent association between atherogenic cholesterol control and class 1 obesity did not reach statistical significance. This may be explained by the lipid panel abnormalities that are seen in obesity. While the lipid profiles of persons with obesity are characterized by LDL-C levels that are normal to slightly elevated, there is a higher proportion of small dense LDL particles among all LDL particles [40]. When compared to larger LDL particles, these smaller, denser LDL particles are more pro-atherogenic as their binding affinity for the LDLR is reduced, which causes higher circulating levels in the blood [40]. These particles also easily infiltrate and are trapped within the arterial wall more easily, which can lead to atherosclerotic plaque formation [40]. Furthermore, second-generation sulfonylureas and long-acting insulin among patients were the second (33.8%) and third (15.5%) most utilized antihyperglycemic medications in the study population. Studies have shown that when sulfonylureas were added to other antihyperglycemic medications in a patient’s regimen, they were associated with a 2.01–2.3 kilograms (kg) weight gain compared to placebo [41]. Insulin also carries the adverse effect of weight gain and studies have demonstrated that insulin-related weight gain ranges between 1.56 and 5.75 kg [41]. Thus, in the case of patients with comorbid T2DM and obesity, the latter might be driving uncontrolled atherogenic cholesterol and medications to treat the former may be contributing to weight gain, which may in turn drive a higher obesity status and perpetuate a deleterious cycle. 

Our study revealed a significant association between patient-level MRC and diabetes-specific MRC. Although we did not evaluate the contribution of the components of the diabetes-specific MRCI score to the overall patient-level MRCI score like Ab Rahman et al., these results seem to suggest that for our study population, diabetes medications and hence diabetes MRC contribute significantly to overall patient-level MRC among patients with low and moderate MRC [15]. Further support for the larger contribution of diabetes medications to the patient-level MRC is threefold. Primarily, there were similar trends in regression analysis results for both patient level and diabetes MRC. Secondly, there was a lower number of patients with comorbid diseases that have inherently complex treatment regimens (asthma, chronic obstructive pulmonary disease, congestive heart failure, chronic kidney disease), so it can be posited that if there were higher percentages of patients with these comorbid conditions in the study population, then the medications treating these conditions would exert more influence on the overall MRC [42,43,44,45,46,47]. Finally, older medication classes have generics that are inexpensive. In a population where most patients have government funded insurance, inexpensive medications are often first-line treatment rather than newer, more efficacious medications, to reduce costs. In the past, pharmacological management of diabetes often meant adding on medications from different antihyperglycemic classes one by one since they each target a different pathophysiological mechanism within the diabetes ominous octet [48]. Resultantly, patients progressively reach increased medication burden, but they may still lack glycemic control. In other words, the overall regimen complexity is being driven up by the number of antihyperglycemic medications and their differing routes, frequencies, and additional directions, but may no longer be effective for glycemic control. 

We found no evidence to support our hypothesis that high diabetes and patient-level MRC would be associated with poor blood pressure control. Intuitively, this hypothesis was born from the idea that regimens with high complexity, whether patient-level or diabetes-specific, would negatively affect patients’ adherence to all medications in their regimens. Thus, blood pressure medication adherence and blood pressure control would be negatively affected by both the overall (patient-level) and diabetes MRC since the latter can be a more complex component of the overall MRC and drive up the score. Our results indicate that there were no associations between blood pressure control and patient level or diabetes MRC at any level (low, moderate, high). Stack et al. characterized the illness perceptions of patients managing co-morbid T2DM, hypertension, and dyslipidemia and found that patients perceived more symptoms and emotional distress in type 2 diabetes than either hypertension or dyslipidemia [49]. One metanalysis that examined the relationship between daily dosing frequency and adherence to antihypertensive medications revealed that once daily dosing regimens were associated with higher rates of adherence when compared to twice daily and multiple daily dosing [50]. Another review examined studies on preference for pharmaceutical treatment process attributes such as dose frequency, route of administration, and dose timing among patients diagnosed and living with diabetes, osteoporosis, autoimmune disorders, or cancer [51]. This review demonstrated that easier or more convenient routes were preferred to difficult ones (oral vs. injections); route administration preference was influenced by treatment frequency; less frequent administration was preferred over more frequent administration; and when compared to timing linked to fixed times such as mealtime, flexible dosing was preferred [51]. Thus, we postulate the following from this supporting literature: patients with T2DM and cardiovascular comorbidities taking multiple medications may experience diabetes-regimen-related distress [32,33,34,49]. As treatment process attributes such as injectable dosage form, more frequent administration, and fixed timing of dosing are less preferred [51], it is plausible that in the face of taking multiple medications for several comorbid chronic conditions, it may be easier to adhere to tablets (most common dosage form for chronic hypertension treatment) at least once daily in comparison to taking multiple tablets and injections (common diabetes treatment dosage forms) multiple times a day. 

There are several limitations of our study. We did not evaluate each patient’s adherence to their overall medication and diabetes medication regimens and as such, we do not know if patients were taking their medications as prescribed at the time of their HbA1c measurement. However, our study aligns with several studies within the literature that have demonstrated that moderate and high diabetes MRC is linked to poor medication adherence [14,15,52]. Since this was a cross-sectional study, the covariates, predictor and outcome variables were all collected based off one point in time, retrospectively. Thus, there are no causal relationships that can be implied between MRC and glycemic control. Yet, the association between MRC and glycemic control or HbA1c has been well documented in the literature through cross-sectional and retrospective cohort analyses [14,15,17,18]. Our study supports these findings and adds to this literature. A noted limitation is bias within the EHR data due to healthcare system practices and processes and patient behavior, which may influence data collection and documentation. More specifically, differing documentation practices and patient behavior can cause data to be missing at random within the EHR. As such, study participants with missing data were not included in the outcome analyses. The medication regimen utilized for the medication regimen complexity analyses were limited to all the medications in the electronic medical record. Since patients with diabetes mellitus regularly see more ambulatory care providers and specialists (primary care, endocrinology, nephrology, cardiology, etc.), fragmented care and thus polypharmacy is common among these patients [53]. Since we did not have access to medications that may have been prescribed by providers outside of the clinical site, or medications that can only be found within patient’s profile at their preferred pharmacy, the MRC scores in this study might be underestimated. Finally, this study has limited generalizability, since it was conducted in one clinic and among non-Hispanic Black patients only. However, given the unique history of structural racism in the United States that has created and maintained racially segregated metropolitan cities that suffer from economic divestment and are characterized as medically underserved and health professional shortage areas, the authors hypothesize that studies conducted in similar areas across the U.S. might yield similar results [54,55,56]. Conducting the study among non-Hispanic Black patients only is also considered a strength of this study as it provides insight on medication-related challenges in a historically marginalized racial group with well-documented disparities in diabetes outcomes. Finally, our study investigated associations between two cardiovascular outcomes that are critical to diabetes management and provides new information regarding diabetes MRC and atherogenic cholesterol and blood pressure control.

## 5. Conclusions

In conclusion, this study reveals that higher levels of diabetes-specific MRC are associated with improved atherogenic cholesterol control, which may be facilitated in part by metformin’s modulatory effects on cholesterol metabolism. However, increasing diabetes-specific MRC correlates negatively with glycemic control, thus presenting a complex challenge in diabetes management. The study also suggests that higher MRC may be a critical component of diabetes distress, which may subsequently lead to poor medication adherence. Pharmacists have collaborated with primary care providers to improve glycemic, atherogenic cholesterol, and blood pressure control in underserved populations for the past two decades [57]. More specifically, within an interdisciplinary model with endocrinologists, pharmacists have co-managed and supported complex patients living with T2DM to achieve glycemic control without increasing patients’ MRC to more than a comparator group of similar patients managed and supported by primary care providers alone [58]. Yet, the integration of pharmacists into population health initiatives and reimbursement for pharmacist services that address population health remains inconsistent across the United States [57]. To help address these challenges, consistent integration of pharmacists into healthcare teams is recommended to optimize therapy plans and ensure a balanced and individualized management of medication regimens. For policy makers, there is a pressing need to reduce the medication burden through improved insurance policies that facilitate access to medications such as SGLT2 inhibitors and GLP-1 receptor agonists, alongside metformin. Further research should extend these observations to include other historically marginalized populations to validate these findings and further explore the impact of social determinants of health on MRC. Such targeted interventions can improve diabetes care and reduce health disparities across diverse communities.

## Figures and Tables

**Table 1:** Sociodemographic Characteristics (N = 470).

Variable	*n* (%)
Gender	
Female	288 (61.3)
Male	182 (38.7)
Age	
Mean ± SD	60.3 ± 12.0
18–54	121 (25.7)
55–64	172 (36.6)
65–74	142 (30.2)
≥75	35 (7.4)
Federal Poverty Level ^1^	
0–99%	375 (79.8)
100–199%	68 (14.5)
≥200%	13 (2.8)
Insurance Status	
Uninsured	37 (7.9)
Insured	433 (92.1)
Insurance Type ^1,2^	
Private	11 (2.3)
Medicaid	308 (65.5)
Medicare	110 (23.4)
My Health LA	8 (1.7)
Other ^3^	18 (3.8)
Housing Status	
Housing Secure	467 (99.4)
Housing Insecure	3 (0.6)
Employment Status ^1^	
Unemployed	310 (66.0)
Employed	72 (15.3)
Retired	33 (7.0)
Smoking Status ^1^	
Never	292 (62.1)
Past	45 (9.6)
Current	111 (23.6)
Alcohol Use ^1^	
No	291 (61.9)
Yes	150 (31.9)

^1^ Summary value N ≠ 470 due to missing data. ^2^ Denotes primary insurance type. ^3^ Predecessor to My Health LA (Healthy Way LA).

**Table 2:** Clinical Characteristics (N = 470).

Variable	*n* (%)
Condition	
Diabetes	
Diet-Controlled	21 (4.5)
Uncomplicated	291 (61.9)
Complicated ^1^	158 (33.6)
Hypertension	392 (83.4)
Dyslipidemia	329 (70)
Obesity (kg/m^2^) ^2,3^	
18.5–24.9	61 (13.0)
25–29.9 (Pre-Obesity)	108 (23.0)
30–34.9 (Class 1 Obesity)	98 (20.9)
35–39.9 (Class 2 Obesity)	76 (16.2)
≥40 (Class 3 Obesity)	85 (18.1)
Coronary Heart Disease	21 (4.5)
Chronic Kidney Disease ^4^	48 (10.2)
Myocardial Infarction	9 (1.9)
Congestive Heart Failure	26 (5.5)
Peripheral Vascular Disease	11 (2.3)
Cerebrovascular Accident or Transient Ischemic Attack	32 (6.8)
Asthma	73 (15.5)
Chronic Obstructive Pulmonary Disorder	24 (5.1)
Charlson Comorbidity Index ^5^	3.6 ± 1.9
Cardiometabolic Measures Overall (units) ^3,5^	
HbA1c (%)	8.4 ± 2.4
SBP (mm Hg)	135.9 ± 19.0
DBP (mm Hg)	81.2 ± 10.7
LDL-C (mg/dL)	109 ± 43.4
HDL-C (mg/dL)	53.3 ± 16.7
TG (mg/dL)	132.3 ± 76.2
TC (mg/dL)	187.1 ± 48.6
BMI (kg/m^2^)	33.4 ± 8.8

^1^ End-organ damage. ^2^ WHO Classification. ^3^ Summary value N ≠ 470 due to missing data. ^4^ Moderate to severe. ^5^ Mean ± standard deviation. Abbreviations: HBA1c: hemoglobin A1c. SBP: systolic blood pressure. DBP: diastolic blood pressure. LDL-C low-density lipoprotein cholesterol. HDL-C: high-density lipoprotein cholesterol. TG: triglycerides. TC: total cholesterol.

**Table 3:** Medication regimen characteristics (N = 470).

Variable	*n* (%)
Patient Level (Overall)	
Number of medications, mean (SD)	8.5 ± 4.7
Patient-level MRCI, mean (SD)	22.4 ± 14.5
Number of medications taken	
≤4	90 (20.2)
5–9	205 (43.6)
10–19	162 (34.5)
≥20	8 (1.7)
Polypharmacy ^1^	
No	91 (19.4)
Yes	379 (80.6)
Patient-level MRCI Categories: tertile ranges	
Low: ≤11.5	115 (25.5)
Moderate: 11.6–31.4	245 (52.1)
High: ≥31.5	110 (23.4)
Number of diabetes medications, mean (SD)	1.8 ± 1.2
Diabetes-specific MRCI, mean (SD)	6.0 ± 4.7
Number of diabetes medications taken	
≤2	368 (78.3)
3–6	102 (21.7)
Diabetes-specific MRCI Categories: tertile ranges	
Low: ≤3	149 (29.6)
Moderate: 4–8	235 (50.0)
High: ≥9	96 (20.4)
Antihyperglycemic Pharmacological Classes	
Sulfonylureas (2nd generation)	160 (33.8)
Meglitinides	1(0.21)
Biguanides	310 (66.4)
Thiazolidinediones	14 (2.8)
Dipeptidyl-peptidase- IV Inhibitors	44 (9.4)
Sodium Glucose co-Transporter 2 Inhibitors	19 (3.8)
Glucagon-Like peptide 1 Receptor Agonists (oral)	1 (0.21)
Glucagon-Like peptide 1 Receptor Agonists (injectable)	4 (0.85)
Combination Medications (oral)	9 (2.1)
Rapid-Acting Insulin	42 (9.0)
Short-acting insulin	61 (13.2)
Intermediate-acting insulin	66 (14.5)
Long-acting insulin	75 (15.5)
Combination Insulin	42 (9.2)
Cardiovascular Medication Use	
Statin Use	258 (54.9)
Aspirin Use	228 (48.5)
Blood Pressure Medication Use ^2^	345 (88.0)

^1^ Polypharmacy defined as taking ≥ 5 medications. ^2^ Total of 392 participants with hypertension diagnosis. Abbreviations: HBA1c: hemoglobin A1c. SBP: systolic blood pressure. DBP: diastolic blood pressure. LDL-C low-density lipoprotein cholesterol. HDL-C: high-density lipoprotein cholesterol. TG: triglycerides. TC: total cholesterol.

**Table 4:** Associations between MRC category and cardiometabolic outcomes.

	Glycemic Control ^1,2^			Atherogenic Cholesterol Control ^1,3^		
Patient Level MRC	Yes, *n* (%)	No, *n* (%)	*p*	Yes, *n* (%)	No, *n* (%)	*p*
Low	34 (43.6)	44 (56.4)	0.041	18 (26.9)	49 (73.1)	0.001
Moderate	82 (37.3)	138 (62.7)		99 (48.3)	106 (51.7)	
High	27 (26.2)	76 (73.8)		55 (56.7)	42 (43.3)	
Diabetes-specific MRC						
Low	67 (67.7)	32 (32.3)	0.000	34 (35.4)	62 (64.6)	0.031
Moderate	65 (31.0)	145 (69.0)		100 (51.8)	93 (48.2)	
High	11 (12.0)	81 (88.0)		38 (47.5)	42 (52.5)	

^1^ Summary value N ≠ 470 due to missing data. ^2^ Glycemic control as binary variable where HBA1c ≥ 7% is uncontrolled and HbA1c < 7% is controlled. ^3^ Atherogenic cholesterol control as binary variable where LDL-C ≥ 100 mg/dL is uncontrolled and LDL-C < 100 mg/dL is controlled Abbreviations: MRC: medication regimen complexity.

**Table 5:** Associations with cardiometabolic outcomes (multiple logistic regression): diabetes MRC.

	Glycemic Control ^1^			Atherogenic Cholesterol Control ^2^		
	AOR	95% CI	*p*	AOR	95% CI	*p*
Diabetes-specific MRC						
Low *	1		<0.001	1		0.104
Moderate	5.329	2.816–10.083	<0.001	0.505	0.266–0.958	0.036
High	23.814	9.023–62.852	<0.001	0.546	0.256–1.165	0.118
Age	0.979	0.952–1.005	0.116	0.979	0.954–1.005	0.117
Gender						
Male *	1			1		
Female	0.983	0.547–1.767	0.955	0.880	0.514–1.504	0.639
Insurance Status						
Uninsured *	1			1		
Insured	0.962	0.232–3.998	0.958	2.090	0.396–11.033	0.385
Employment Status						
Unemployed *	1		0.271	1		
Employed	1.861	0.863–4.014	0.113	1.060	0.541–2.078	0.865
Retired	1.323	0.466–3.754	0.599	0.973	0.360–2.629	0.957
Alcohol Use						
No *	1			1		
Yes	1.236	0.670–2.281	0.497	1.886	1.074–3.311	0.027
Smoking Status						
Never *	1		0.953	1		
Past	0.967	0.377–2.482	0.944	0.817	0.350–1.905	0.639
Current	0.891	0.429–1.849	0.756	0.865	0.447–1.677	0.668
Federal Poverty Level						
0–99% *	1		0.629	1		
100–199%	0.819	0.382–1.753	0.607	1.387	0.683–2.814	0.365
≥ 200%	2.582	0.235–28.350	0.438	1.425	0.327–6.205	0.637
WHO BMI Groups						
18.5–24.9 *	1		0.448	1		
25–29.9 Pre-Obesity	2.279	0.911–5.700	0.078	3.209	1.298–7.934	0.012
30–34.9 Class 1 Obesity	1.489	0.603–3.680	0.388	2.330	0.936–5.800	0.069
35–39.9 Class 2 Obesity	1.212	0.460–3.194	0.697	3.350	1.278–8.780	0.014
≥ 40 Class 3 Obesity	1.461	0.544–3.919	0.452	3.310	1.255–8.729	0.016
Charlson Comorbidity Index	1.054	0.913–1.216	0.472	0.976	0.856–1.112	0.711

^1^ Glycemic control as binary variable where HBA1c ≥ 7% is uncontrolled and HbA1c < 7% is controlled. ^2^ Atherogenic cholesterol control as binary variable where LDL-C ≥ 100 mg/dL is uncontrolled and LDL-C < 100 mg/dL is controlled. * Reference. Abbreviations: MRC: medication regimen complexity. AOR: adjusted odds ratio.

**Table 6:** Associations with cardiometabolic outcomes (multiple logistic regression): patient-level MRC.

	Glycemic Control ^1^			Atherogenic Cholesterol Control ^2^		
	AOR	95% CI	*p*	AOR	95% CI	*p*
Patient-Level Specific MRC						
Low *	1		0.010	1		0.009
Moderate	1.959	0.977–3.930	0.058	0.396	0.172–0.916	0.030
High	3.625	1.572–8.358	0.003	0.230	0.090–0.592	0.002
Age	0.976	0.951–1.001	0.058	0.981	0.955–1.007	0.146
Gender						
Male*	1			1		
Female	1.098	0.644–1.870	0.732	0.912	0.530–1.567	0.738
Insurance Status						
Uninsured *	1			1		
Insured	0.741	0.200–2.750	0.655	1.789	0.331–9.650	0.499
Employment Status						
Unemployed *	1		0.150	1		0.773
Employed	2.025	0.978–4.193	0.057	0.855	0.430–1.701	0.655
Retired	1.388	0.530–3.635	0.505	0.715	0.250–2.052	0.533
Alcohol Use						
No *	1			1		
Yes	1.401	0.799–2.454	0.239	1.761	0.995–3.117	0.052
Smoking Status						
Never *	1		0.708	1		0.970
Past	0.733	0.318–1.691	0.467	0.954	0.407–2.235	0.914
Current	1.090	0.565–2.103	0.796	0.922	0.473–1.799	0.812
Federal Poverty Level						
0–99% *	1		0.579	1		0.733
100–199%	0.978	0.483–1.979	0.950	1.387	0.683–2.814	0.556
≥200%	3.097	0.366–26.223	0.300	1.425	0.327–6.205	0.559
WHO BMI Groups						
18.5–24.9 *	1		0.330	1		0.051
25–29.9 Pre-Obesity	2.014	0.868–4.676	0.103	3.226	1.290–8.065	0.012
30–34.9 Class 1 Obesity	1.409	0.625–3.178	0.408	2.270	0.903–5.704	0.081
35–39.9 Class 2 Obesity	0.929	0.388–2.227	0.869	3.885	1.454–10.380	0.007
≥40 Class 3 Obesity	1.281	0.521–3.146	0.589	3.816	1.420–10.258	0.008
Charlson Comorbidity Index	1.058	0.926–1.209	0.407	1.008	0.800–1.154	0.908

^1^ Glycemic control as binary variable where HBA1c ≥ 7% is uncontrolled and HbA1c < 7% is controlled. ^2^ Atherogenic cholesterol control as binary variable where LDL-C ≥ 100 mg/dL is uncontrolled and LDL-C < 100 mg/dL is controlled. * Reference. Abbreviations: MRC: medication regimen complexity. AOR: adjusted odds ratio.

## Data Availability

Data are contained within the article.

## Appendix A

**Table A1:** Cardiometabolic outcomes (N = 470).

Variable	*n* (%)
Glycemic Control ^1,2^	
Controlled	143 (30.4)
Uncontrolled	258 (54.9)
Atherogenic Cholesterol Control ^1,3^	
Controlled	172 (36.6)
Uncontrolled	197 (41.9)
Systolic Blood Pressure Control ^1,4^	
Controlled	291 (61.9)
Uncontrolled	169 (36.0)
Diastolic Blood Pressure Control ^1,5^	
Controlled	370 (78.7)
Uncontrolled	90 (19.1)

^1^ Summary value N ≠ 470 due to missing data. ^2^ Glycemic control as binary variable where HBA1c ≥ 7% is uncontrolled and HbA1c < 7% is controlled. ^3^ Atherogenic cholesterol control as binary variable where LDL-C ≥ 100 mg/dL is uncontrolled and LDL-C < 100 mg/dL is controlled. ^4^ Systolic blood pressure control as binary variable where SBP ≥ 140 mm Hg is uncontrolled and SBP < 140 mm Hg is controlled. ^5^ Diastolic blood pressure control as binary variable where DBP ≥ 90 mm Hg is uncontrolled and DBP < 90 mm H is controlled.

**Table A2:** Associations between diabetes MRC category and diabetes mellitus severity.

	Diabetes Mellitus Severity ^1^					
Diabetes-Specific MRC	Diet-Controlled *n* (%)		Uncomplicated *n* (%)		Complicated *n* (%)	
		*p*		*p*		*p*
Low	21 (100.0)	<0.001	89 (30.6)	<0.001	29 (18.4)	<0.001
Moderate	0		148 (50.9)		87 (55.1)	
High	0		54 (18.6)		42 (26.6)	

^1^ Summary value N: 470; Abbreviations: MRC: medication regimen complexity.
